# Supplementary figures and images for: CSMD1 Mutations Are Associated with Increased Mutational Burden, Favorable Prognosis, and Anti-Tumor Immunity in Gastric Cancer
Source: Genes (Basel). 2021 Oct 28;12(11):1715. doi: 10.3390/genes12111715 (PMC8623648; doi:10.3390/genes12111715)

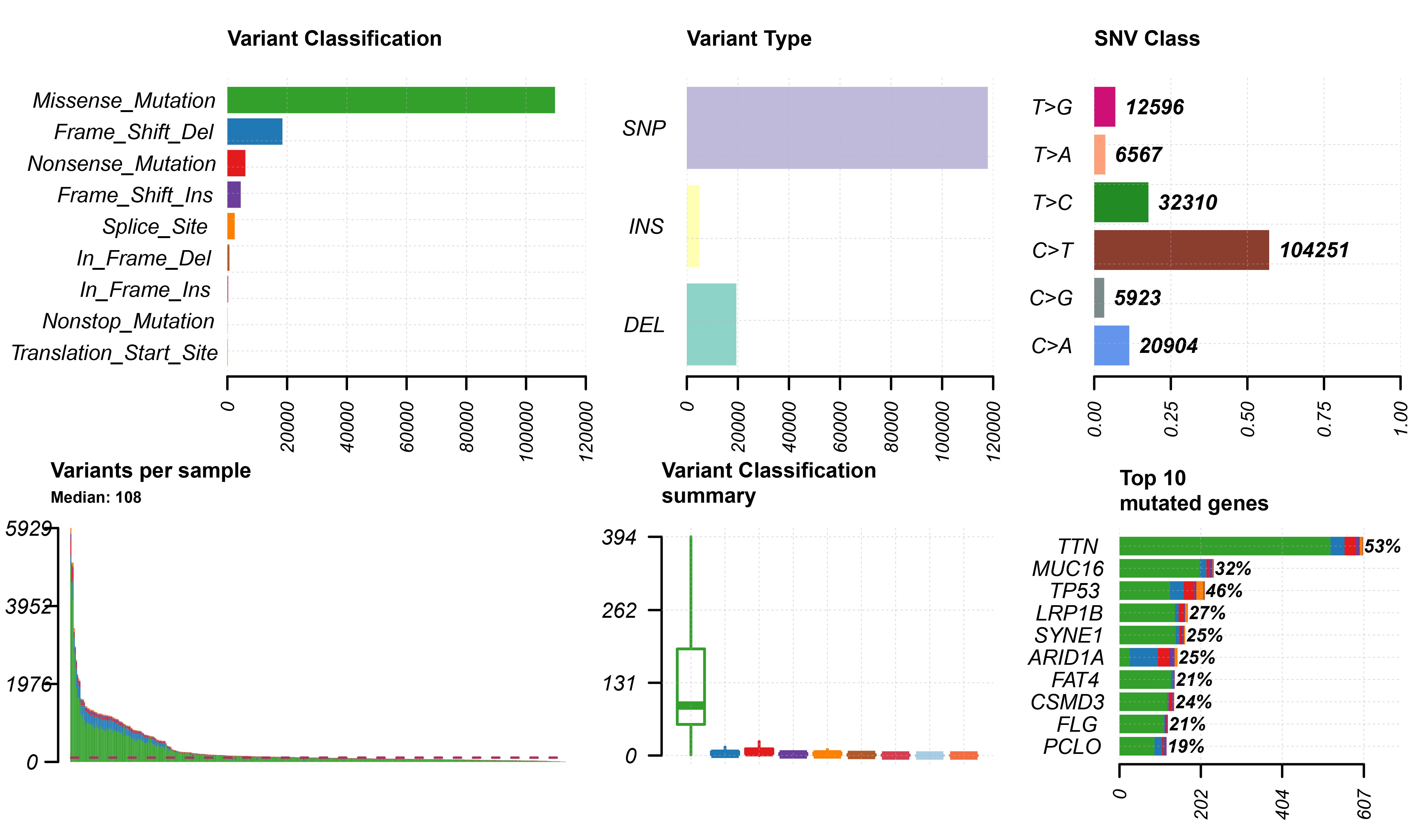

Supplement: Supplementary file 1 [file genes-12-01715-s001.zip › genes-1441798-supplementary Figure S1.jpg]

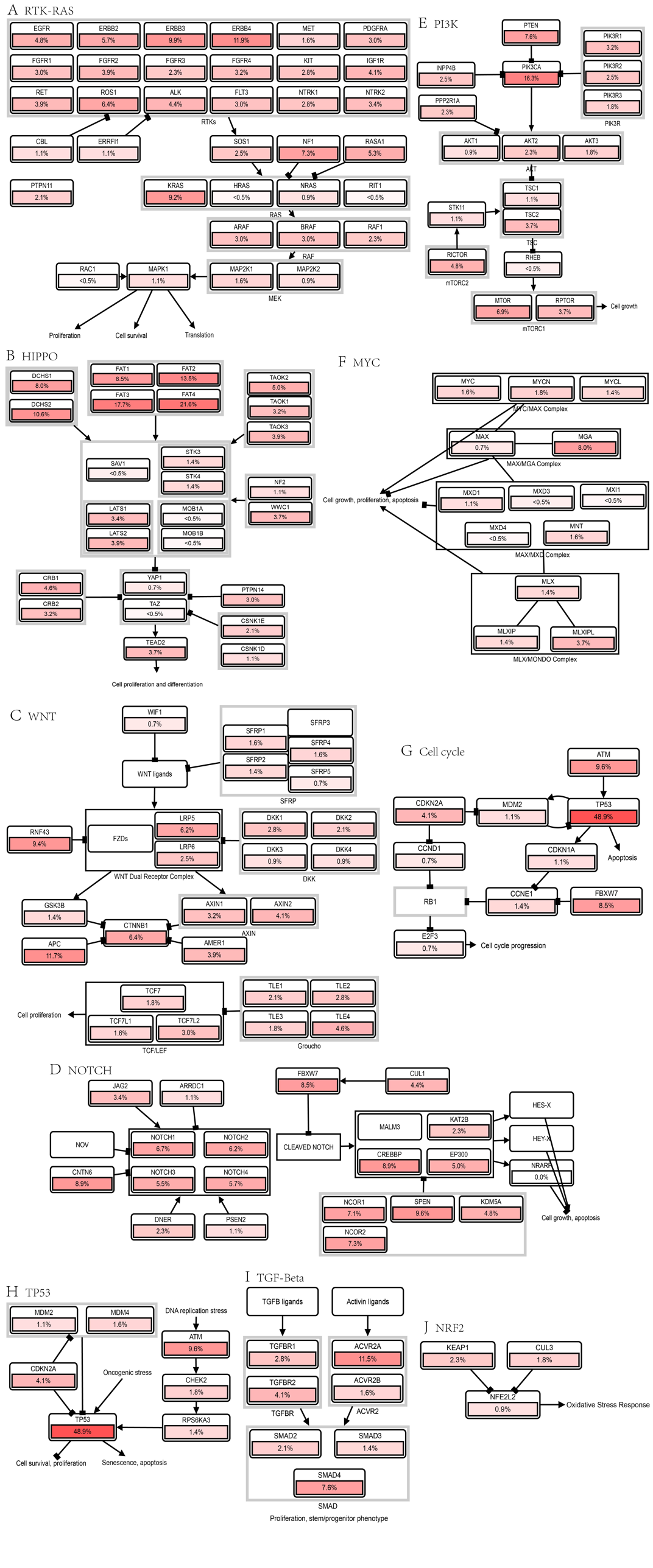

Supplement: Supplementary file 1 [file genes-12-01715-s001.zip › genes-1441798-supplementary Figure S2.jpg]
